# Supplementary material for: Using an algorithmic approach to shape human decision-making through attraction to patterns
Source: Nat Commun. 2025 May 2;16:4110. doi: 10.1038/s41467-025-59131-4 (PMC12048589; doi:10.1038/s41467-025-59131-4)
Supplement: Supplementary file 1 — Supplementary Information [file 41467_2025_59131_MOESM1_ESM.pdf]

Supplementary Information

Title:
Using an algorithmic approach to shape human decision-making through attraction to patterns

Supplementary Discussion:
Overview
A qualitative description of the underlying elements of schedules SS2 and SS4
Results

Coda: Decision making in regular and irregular (reward) environments

Supplementary Methods:
Description of the RaCaS algorithm
Exceptions and changes in transitions between stages

Supplementary Figures S1-S6:
Supplementary Figure 1: *Comparison of the two nearly -identical versions of the RaCaS algorithm*
Supplementary Figure 2: *Under RaCaS, participants discovered more rewards on the Bias+ side, and fewer rewards on the Bias- side*
Supplementary Figure 3: *Empirically observed reward expectancy for both sides, as observed by participants assigned to RaCaS and to all other algorithms.*
Supplementary Figure 4: *Participants in general and RaCaS participants in particular, exhibited longer response times when choosing the Bias- option*
Supplementary Figure 5: *Assessing the biasing efficacy of different elements in RaCaS and in SS2/4: "First impression matters" and "If it ain't broke, don't fix it"*
Supplementary Figures 6: Assessing the biasing efficacy of different elements in RaCaS and in SS2/4: "Learned helplessness" and "The gamblers fallacy"

Supplementary Data 1:
CEC's Statistics

Supplementary References

Supplementary Notes

Overview

This supplementary text serves two main goals: The first is to isolate, as best as possible, the effect that RaCaS' unfolding sequence of rewards has on biasing participants' choice-behavior. The second goal is to assess the efficacy of other design elements for engineering choices, in the context of the Choice Engineering Competition (CEC[1]).

We advance both goals by focusing on three elements designed to influence participants' choice in the two schedules we submitted to the CEC's 'static track' (SS02 and SS04, as named by the competition's organizers[2], hereafter referred to as SS2, SS4 or SS2/4 when mentioned together). Compared to the RaCaS algorithm, which was designed to dynamically respond to participants' behavior, the measurement of the components inserted for influencing choice-behavior in SS2/4 is straightforward, due to their 'static' nature. These schedules consisted of a fixed list for reward allocation, which was shared by all participants regardless of their specific choices (see Supplementary Figure 1 for both SS2/4 schedules).

Contrasting the effects of potentially-biasing elements in SS2/4 with the effect the same elements had on RaCaS' participants, provides further information on the effect RaCaS' unfolding sequence had on participants' choices. Specifically, a decrease in the biasing influence of these elements in RaCaS or a difference in their dynamics (while maintaining an average bias that is at least as strong in RaCaS as that generated by the other two schedules), can be plausibly credited to the elements unique to RaCaS, and foremost, to its implementation of an unfolding sequence.

In what follows, we first describe the principles that guided the design of SS2/4. Then, we report the empirical effectiveness for each a-priori element in SS2/4 and in RaCaS. We thus present the results from two different angles, answering two different questions: 1-What do they teach us about the effectiveness of the said elements in the CEC task? 2- What does a difference between the effectiveness of these elements in SS2/4 and in RaCaS teach us about RaCaS' unique properties.

A qualitative description of the underlying elements of schedules SS2 and SS4

1. **"First impression matters"**. Early rewards have an increased impact on subsequent choices. Therefore, SS2 and SS4 were designed to maximize the likelihood of discovering rewards in $Bias^+$, while minimizing the likelihood of discovering rewards in $Bias^-$, early in the experiment.
2. "**If it ain't broke, don't fix it**". People will stick with their choice as long as it is rewarded. In light of this principle, in both SS2 and SS4, rewards in $Bias^+$ were allocated in continuous sequences. The same idea was used in SS2/4 in an attempt to 'hide' rewards that were obligatorily allocated to $Bias^-$, by lagging them after the initiation of

the $Bias^+$ reward sequences, assuming that participants would be less likely to explore the $Bias^-$ side immediately after being rewarded on the $Bias^+$ side.

3. **"Learned helplessness".** We predicted that after repeated failed attempts to attain rewards for a specific choice, people will stop exploring the seemingly unprofitable option. To implement the above principle and in tandem with principle #1 and #2, residual rewards designated to $Bias^-$ were allocated late in the experiment. We hypothesized that individuals would acquire enough failures to obtain rewards when choosing the $Bias^-$ option throughout the task, such that when, in later trials, these residual rewards are in fact abundantly allocated to the $Bias^-$ option, participants will not explore that option.

Supplementary Results

All reported statistical tests are two-tailed if not mentioned otherwise. In general, both SS2 and SS4 successfully led participants towards favoring option $Bias^+$, SS2: 64.1% and SS4: 61%. In fact, SS2 biased participants in a manner that is not significantly different than the reported winner of the 'static track' of the CEC, 64.1% versus 64.3% ($t(1131)=0.2$, $p=0.84$, two-sample t-test[2]). The fact that SS2/4 successfully biased choice-behaviour, provides an initial validation of the (combined) efficacy of their underlying principles. Compare this to the chance level of 50% and to the average bias generated by RaCaS, the best performing algorithm in both tracks of the CEC, which was 70%.

"First impression matters". In order to assess the differential impact of early trials in determining participants' further choice behavior, we correlated the number of $Bias^+$ rewards received during the early trials of the experiment with the participant's overall bias ('early' was defined as the first 15 trails - the minimal window in which a sufficiently large number of rewards is allocated in both SS2 and SS4, see Supplementary Figure 5A). Results show (see Supplementary Figure 5B top) that, as predicted, the number of rewards received for $Bias^+$ choices during the first 15 trails was indeed (strongly) correlated with the total bias (towards $Bias^+$), for both SS2 and SS4, with Pearson's coefficient values of $r(545)=.611$ and $r(206)=.678$, correspondingly (SS2; $p<.001$, SS4; $p<.001$). For RaCaS, the same correlation was too, significant ($r(260)=0.437$, $p<.001$), but also significantly smaller than that of SS2/4 (RaCaS vs. SS2; $z=3.192$, $p<.0015$. RaCaS vs. SS4; $z=3.81$, $p<.001$. Fisher Z transformation followed by a Z-test, comparing two correlation coefficients), suggesting that while first impression matters in RaCaS, other dominant elements may overshadow its effect. Note that the confirmation of the above design principle is far from intuitive. In fact, prominent reinforcement learning models consider the most recent rewards are having the greatest impact, with their effect decreasing exponentially with the distance from the current trial[3].

However, these results should be interpreted with caution, due to their correlational nature. Specifically, it is difficult to determine whether finding more $Bias^+$ rewards during the first 15 trials led to an increase in the bias towards this side, or, alternatively and somewhat trivially, that

participants with a stronger bias towards Bias$^+$ found more rewards on this side just because they chose it more. Conversely, using a relatively small proportion of the experiment's early trials for this analysis somewhat weakens this alternative explanation. To further test whether rewards received early on have a larger effect on participants' total bias than their sheer amount, we compared the above correlation to that between the total bias and 15 trials randomly sampled from trials 16-100 (see Supplementary Figure 5B bottom).

The correlation calculated between the rewards won in the 15 random trials and the total bias was less than half of that between the first 15 trials and total bias for SS2 (SS2 first 15 $r(545)=.611$ vs random 15 $r(545)=0.257$; $z = 7.37$, $P<0.001$. Fisher Z transformation followed by a Z-test, comparing two correlation coefficients) and was significantly reduced also for SS4 (SS4 first 15 $r(206)=0.678$ vs random 15 $r(206)=0.433$; $z =3.6625$, $P<0.001$. Fisher Z transformation followed by a Z-test, comparing two correlation coefficients), further establishing the unique role early wins play in both schedules.

The results for RaCaS' participants are very different; the correlation was stronger for the 15 randomly sampled trials (RaCaS' first 15 $r(0.437)$ vs random 15 $r(0.584)$; $z =2.268$, $p=0.023$. Fisher Z transformation followed by a Z-test, comparing two correlation coefficients), suggesting that in RaCaS, unlike SS2/4, this element has no measurable effect.

To conclude – while early rewards can be over weighted in the context of decision making, RaCaS produces a bias that seems to be independent of this influence. As will become clear, this is the first of the findings, which together, suggest that under RaCaS, participants' choice behavior is, in general, less sensitive to rewards and their dynamics, other than those directly involved in the regular sequence. However, we note that a more rigorous way to address this question in the future would involve a controlled manipulation of the number of rewards received by participants during the initial stages of the experiment and a subsequent measurement of the manipulation's impact on their final bias.

"If it ain't broke, don't fix it". This design principle dictates that on trial t +1 participants will tend to stick with the choice they made on trial t, given that it was rewarded. To empirically assess whether this was indeed the case, we first look at participants' behavior under each schedule, regardless of whether it was rewarded or not. Participants were generally more likely to stay with their previous choice, regardless of its outcome, with this pattern most strongly observed for RaCaS' participants (Supplementary Figure 5C, left). Yet, as predicted, when the probability of staying with the same choice is conditioned on whether the choice followed a rewarded or unrewarded choice (see Supplementary Figure 5C, right), the probability that participants under SS2/4 and RaCaS stay with the same option, is significantly larger when they have just received a reward than when they did not receive a reward [RaCaS; 19175 no reward trials vs. 6501 reward trials, $p<0.001$. SS2; 40124 no reward trials vs. 13482 reward trials, $p < 0.001$. SS4; 15487 no reward trials vs. 4897 reward trials, $p< 0.\ 001$. Fisher exact tests]. However, this difference was smaller in RaCaS, showing, yet again, the reduced effect that

rewards have on RaCaS participants' choice behavior. Specifically, the increased tendency of RaCaS participants to stick with their choice in those cases where they were not rewarded, suggests that the sequence in RaCaS fosters loyalty to $Bias^{+}$ beyond immediate reward events.

"Learned helplessness". To lower participants' expectancy of receiving a reward when choosing the $Bias^{-}$ option, rewards allocated to the $Bias^{-}$ side were hidden for as long as possible. The efficacy of this principle for generating bias was empirically assessed by correlating the time (trial) of first discovery of a $Bias^{-}$ reward and the total bias. The results show that this worked as predicted in SS2/4, with time of discovery of the first reward on the $Bias^{-}$ side strongly and positively correlated with the number of choices of the $Bias^{+}$ option (SS2; $r(511)=0.497$, $p<0.001$. SS4; $r(184)=0.491$, $p<0.001$.). However, this principle does not seem to operate in RaCaS, where the timing of discovery of a first reward in option $Bias^{-}$ was not noticeably correlated with participants' total bias ($r(182)=0.089$, $p=0.229$, Supplementary Figure 6A). This is yet another testimony that while in SS2/4 participants' choice-behavior is highly sensitive to the reward schedules of both options, RaCaS lowers exploration of the $Bias^{-}$ option mostly through the regularity associated with the $Bias^{+}$ option, thus minimizing the impact of other factors. Once again, we note that future experiments may allow a more careful assessment of these findings, such as the random allocation of participants to groups manipulated to first discover rewards on the $Bias^{-}$ side early vs. late in the experiment.

Coda: Decision making in regular and irregular (reward) environments

Another way to interpret the seeming lack of sensitivity of RaCaS' participants to rewards and reward schedules, is that much of the behavior that is successfully captured by popular reinforcement learning models occurs in random environments, highly probabilistic ones, or more generally, in environments that are not (subjectively) well understood by the agent. The flipside of this is that when the environment is structured (or has been subjectively understood by the agent as such) as is the case for option $Bias^{+}$ in RaCaS, reinforcement learning models may be less relevant for predicting behavior.

This varying extent of subjective 'understanding' of the different environments may be reflected in another, unpredicted, finding, which resembles a Gambler's fallacy-like behavior[4]; as can be seen in Supplementary Figure 6B, participants under SS2/4 show a near linear positive relationship between the probability of re-selecting the same option (Staying) and the sequence of unrewarded selections of that option. In other words, under these schedules, participants tend to re-select an option more, the more consecutive trials it failed to reward them, as if it 'owes' them rewards. Notably, this finding again appears incongruous with the dominant reinforcement learning paradigm, which predicts a decrease in the value associated with some option following a (repeated) failure to obtain reward when selecting that option[3] (and thus a decreased chance it will be chosen). Under RaCaS, the pattern displayed by participants is more complex, with the same increasing pattern observed in SS2/4 appearing only after four unrewarded selections occurred.

Intriguingly, in RaCaS, more than four non-rewarded clicks occurred only when participants experienced a sequence that was broken/non-existent – or in other words, an environment that was experienced (at least, transiently) as random. Likewise, we suggest that this was the constant, or at least dominant, perception under both SS2/4.

Given the above, we suggest that the Gambler's fallacy can (ironically) serve as another index to participants' subjective perception of how stochastic a reward-environment is, with the fallacy kicking in mostly in environments perceived as being stochastic. Future experiments, in which the subjective feeling of 'understanding the environment' is directly measured for each participant, may be valuable in putting the above suggestion to a better test.

Supplementary Methods

Description of the RaCaS algorithm:

What follows is a verbal description of the RaCaS algorithm, the exact procedure is dictated by the publicly available Python code, which can be found here: https://github.com/orena1/Specious_Regularity

Rewards on Bias$^+$:

1. A reward is assigned to the Bias$^+$ side contingent on a single unrewarded click on Bias$^+$ (such that if the participant will sequentially click on the Bias$^+$ option, the first click will not be rewarded, and the second one will be).
   This OFF-ON reward schedule (no-reward, reward, no-reward, reward, etc.) lasts for the first 10 trails of the experiment, regardless the participant's choices; we name this phase 'Stage 0' and assign a value for the parameter, X=1, for the number of clicks required before a reward is allocated.
2. If at least one reward is collected during the first 11 trials, the next phase would begin on the 12th trial, such that now two consecutive clicks on the Bias$^+$ option are required for a reward to be allocated (i.e. every 3rd click is rewarded), this stage (Stage 1, X=2), continues until trial 25. By default, the number of required consecutive clicks (X) changed according to a predetermined timeline. This timeline was chosen (somewhat intuitively) to ensure that regularity is present for most participants throughout most of the 100 trials, given the task constraints (i.e. 25 rewards per option over 100 trials). The decision to cap X at four stemmed from the wish to avoid rewarding frequencies that are too sparse, which may lead to exploration of the alternative option. Similarly, having X increase and then decrease was meant to counter a potential decrease in the utility associated with the Bias$^+$ option (due to the constant increase in the effort required to obtain it without a comparable increase in the associated outcomes). The Bias$^+$ sequence's unfolding is thus:

Stage 0: $1 \leq$trial ≤ 11, X=1
Stage 1: 12 $\leq$trial$\leq$25, X=2
Stage 2: 26 $\leq$trial$\leq$40, X=3
Stage 3: 41 $\leq$trial$\leq$60, X=4
Stage 4: 61 $\leq$trial$\leq$80, X=3
Stage 5: 81 $\leq$trial$\leq$100, X=2

3. When the necessary number of consecutive clicks for receiving a reward is not met (Because a participant chose $Bias^-$ one or more times), the same number of necessary-consecutive clicks needs to be achieved upon returning to the $Bias^+$ option, unless a stage transition occurred.
4. The number of clicks required for the allocation of a reward is dictated by the position of the last required OFF trial within the scheme above, and not by the position of the first trial in the sequence. For example, if one starts clicking sequentially on the $Bias^+$ on trial 39 (which places it in the range of X=3), they will receive a reward at 43 (rather than 42), as once the trial>40, X is set to 4, increasing the necessary number of clicks by 1 (i.e., Stage 3 with X=4 will kick in when trial>40). Conversely, if one starts clicking sequentially on the $Bias^+$ at trial 38 (which places it in the range of X=3), they will receive a reward at 41, as the last required OFF choice takes place on trial 40, just before the stage transition occurs.
5. Excess $Bias^+$ rewards are left to the last trials of the experiment. This implies that if the number of remaining trials is equal to the number of remaining rewards, a reward would be allocated at every trial until the end.

Rewards on $Bias^-$:

Allocation of rewards to the $Bias^-$ side begins only when the following conditions are met:

- The experiment reached Stage 1 for the first time and the previous $Bias^+$ reward was gained: a first reward at $Bias^-$ will be allocated simultaneously with the next $Bias^+$ reward, as long that the participant continues choosing the $Bias^+$ option sequentially.
- In all later stages, rewards at the $Bias^-$ option occur simultaneously with the allocation of a reward to $Bias^+$ (or with the was-to-be-allocated $Bias^+$ reward, in cases where the $Bias^+$ rewards were depleted before the experiment had ended)
- Excess $Bias^-$ rewards are left to the last trials of the experiment. This implies that if the number of remaining trials is equal to the number of remaining rewards, a reward would be allocated at every trial until the end.

The $Bias^-$ complete allocation scheme is thus:

Stage 0: ($1 \leq$trial ≤ 11, X=1): No rewards are allocated at the $Bias^-$ option
Stage 1: (12 $\leq$trial$\leq$25, X=2): Rewards are allocated at $Bias^-$ simultaneously with $Bias^+$, but only after the participant's history contains at least one $Bias^+$ reward while $2 \leq X$

Stage 2: (26 $\leq$trial$\leq$40, X=3): Rewards are allocated at Bias$^-$ simultaneously with Bias$^+$
Stage 3: (41 $\leq$trial$\leq$60, X=4): Rewards are allocated at Bias$^-$ simultaneously with Bias$^+$
Stage 4: (61 $\leq$trial$\leq$80, X=3): Rewards are allocated at Bias$^-$ simultaneously with Bias$^+$
Stage 5: (81 $\leq$trial$\leq$100, X=2): Rewards are allocated at Bias$^-$ simultaneously with Bias$^+$

Exceptions and changes in transitions between stages:

- Adjusting the starting point of the scheme(t10): If the participant chose only the Bias- during the first 10 trials of the experiment, the scheme's activation point was pushed forward by 10 trials as was the shift between phases. This point was generalized, such that the starting point was set to be the nearest ten from below:

  Start = 10*(int(First_b+ /10))

  In practice, this adjustment never occurred.

- Schedule reset: Starting from the 12th trial, If the model detects 10 sequential clicks (regardless of which option is selected, or the mix of such choices) without any rewarded Bias$^+$ choice, the schedule resets. The addition of schedule reset was meant to fulfill two goals: First, to ease learning of the pattern by reducing the number of consecutive clicks required to receive a reward. Second, to allow a more efficient allocation of rewards, assuming that rewards are not allocated often enough due to the participant's failure to activate the sequence.
  When designing DS1 and DS2, we experimented with different reset implementations (this was, in fact, the only place where the two versions that constitute RaCaS were meant to be different. In practice, however, the difference in design did not translate to real empirical difference; i.e. when participants' actual choices are fed into the two different versions, they yield identical outputs). Our initial observations demonstrated that the exact implementation may not be very important, as long as the two goals stated above are somehow promoted, that is – the difficulty of discovering the sequence is lowered and the allocation of rewards becomes more frequent following a prolonged failure to win rewards when choosing the Bias$^+$ option. While we experimented with more sophisticated (adaptive per participant) methods for resetting the schedule, in the versions we submitted to the CEC, the reset activated a much simpler process: following a reset, the number of trials left until the end of the experiment was evaluated. Then, the first 10% of these trials were assigned with Stage 0 (X=1), and all other trials were assig ned with Stage 1 (where X=2). While this exact implementation does not fully capture the design originally conceived, it does satisfy both purposes of the reset: The complexity of the sequence is dramatically reduced, and the frequency of rewards increases. Having said that, it was not adaptive to different participants' decision-making behavior and thus often led to a non-optimal reward allocation following schedule reset (See Figure 1, Participant #9, observe the absence of Bias$^+$ rewards in the last ~20 trials.

Reset occurs at trial 54). Further research and experimentation with variations on RaCaS, could assess the efficiency of other reset methods and parametrization.

Supplementary Figures

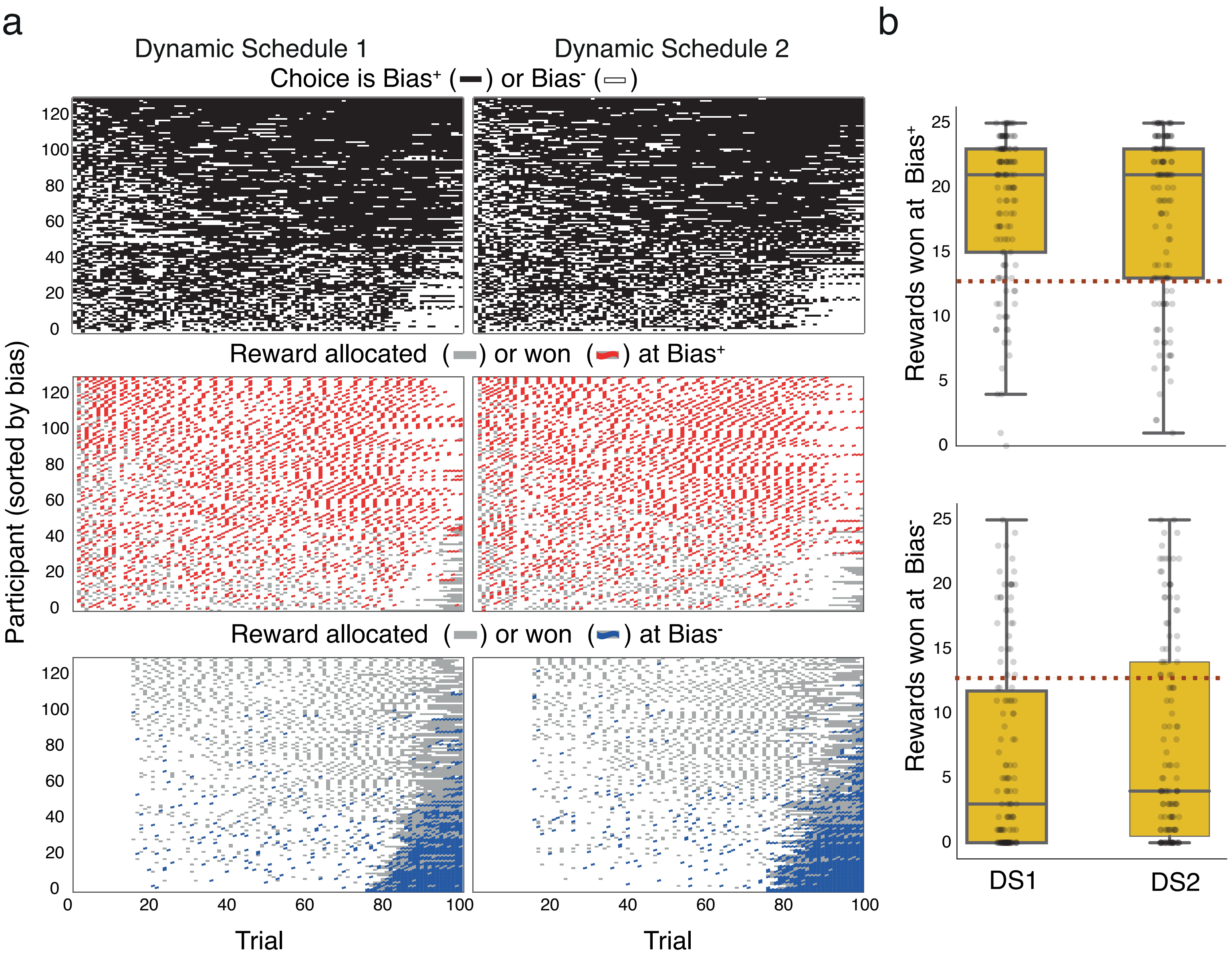

Supplementary Figure 1. *Comparison of the two nearly -identical versions of the RaCaS algorithm.* (A) Top: Raster plot depicting all participants' choices across all 100 trials (the full dataset, ordered by the magnitude of bias, with higher rows indicating more biased participants). The small black bar depicts a choice of Bias+ and a small white bar, a choice of Bias-. Middle: Raster plot depicting discovery of the rewards for the Bias+ option in both algorithms across all 100 trials. Participants were ranked by magnitude of their bias, with strongest at the top. Bottom: Same as above, but for the Bias- option. (B) Boxplots depicting the sum of rewards gained on the Bias+ side (top) and Bias- side (bottom) by participants allocated to both algorithms. Dots indicate individual participants. The dashed brown line indicates the expected value under random choice. The differences were not significant between both the Bias+ ($t(260)=0.602$, $p=0.547$, 95% CI: -1.03-1.95) and the Bias- ($t(260)=-1.06$, $p=0.287$, 95% CI: -2.912-0.866) sides (two-sample *t*-tests). Box bounds show interquartile range (IQR), line represent the median, and whiskers extend to points that lie within 1.5 IQRs.

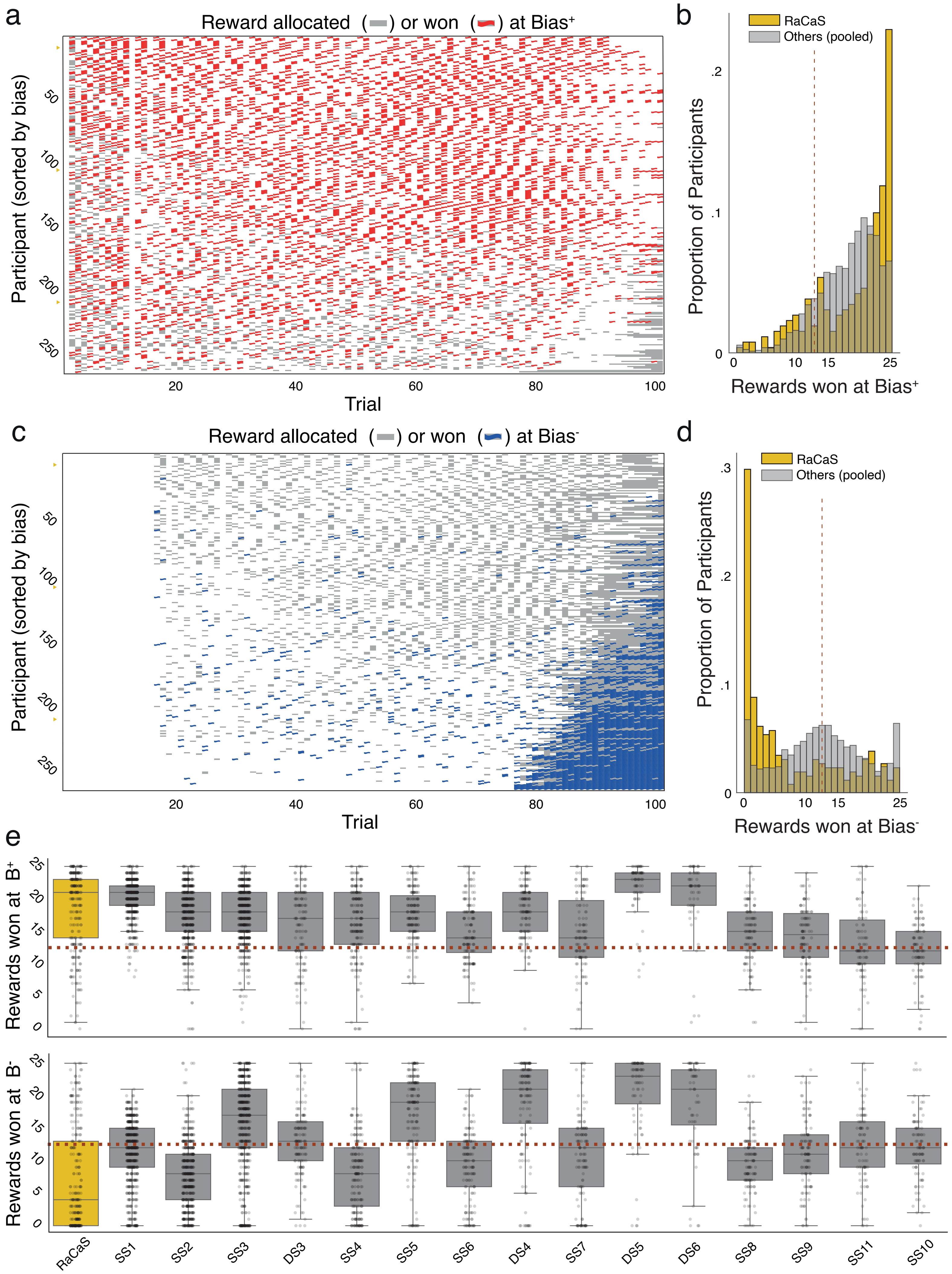
a
Reward allocated ( ) or won ( ) at Bias+
Participant (sorted by bias)
50
100
150
200
250
20
40
60
80
100
Trial
b
RaCaS
Others (pooled)
Proportion of Participants
.2
.1
0
0
10
15
25
Rewards won at Bias+
c
Reward allocated ( ) or won ( ) at Bias-
Participant (sorted by bias)
Trial
d
RaCaS
Others (pooled)
Proportion of Participants
.3
.2
.1
0
Rewards won at Bias-
e
Rewards won at B+
Rewards won at B-
25
20
15
10
5
0
RaCaS
SS1
SS2
SS3
DS3
SS4
SS5
SS6
DS4
SS7
DS5
DS6
SS8
SS9
SS11
SS10

Supplementary Figure 2. *Under RaCaS, participants discovered more rewards on the Bias+ side, and fewer rewards on the Bias- side.* (A) A raster plot depicting RaCaS reward allocation (small grey bar) in all experiments, across all 100 trials (the full dataset, ordered by strength of bias, where higher indicates more bias). Red tilde indicates a reward discovered by a participant. (B) Two overlaid histograms depicting the proportion of participants collecting different numbers of Bias+ allocated rewards, with 0 denoting the minimal possible quantity and 25 the maximum. RaCaS performance is in yellow and all other competing algorithms in grey. The red dashed line depicts the expected number of rewards for a randomly behaving agent. (C) Same as A, but for the Bias- side. (D) Same as B, but for the Bias- side. (E) Boxplots depicting the number of rewards obtained on the Bias+ side (top) and the Bias- side (bottom) by participants under RaCaS (yellow) and under all other algorithms (grey). Dots represent individual participants. Algorithms are ranked by magnitude of Bias+ they produced. The dashed brown line depicts the expected number of rewards under random choice. Box bounds show interquartile range (IQR), line represent the median, and whiskers extend to points that lie within 1.5 IQRs.

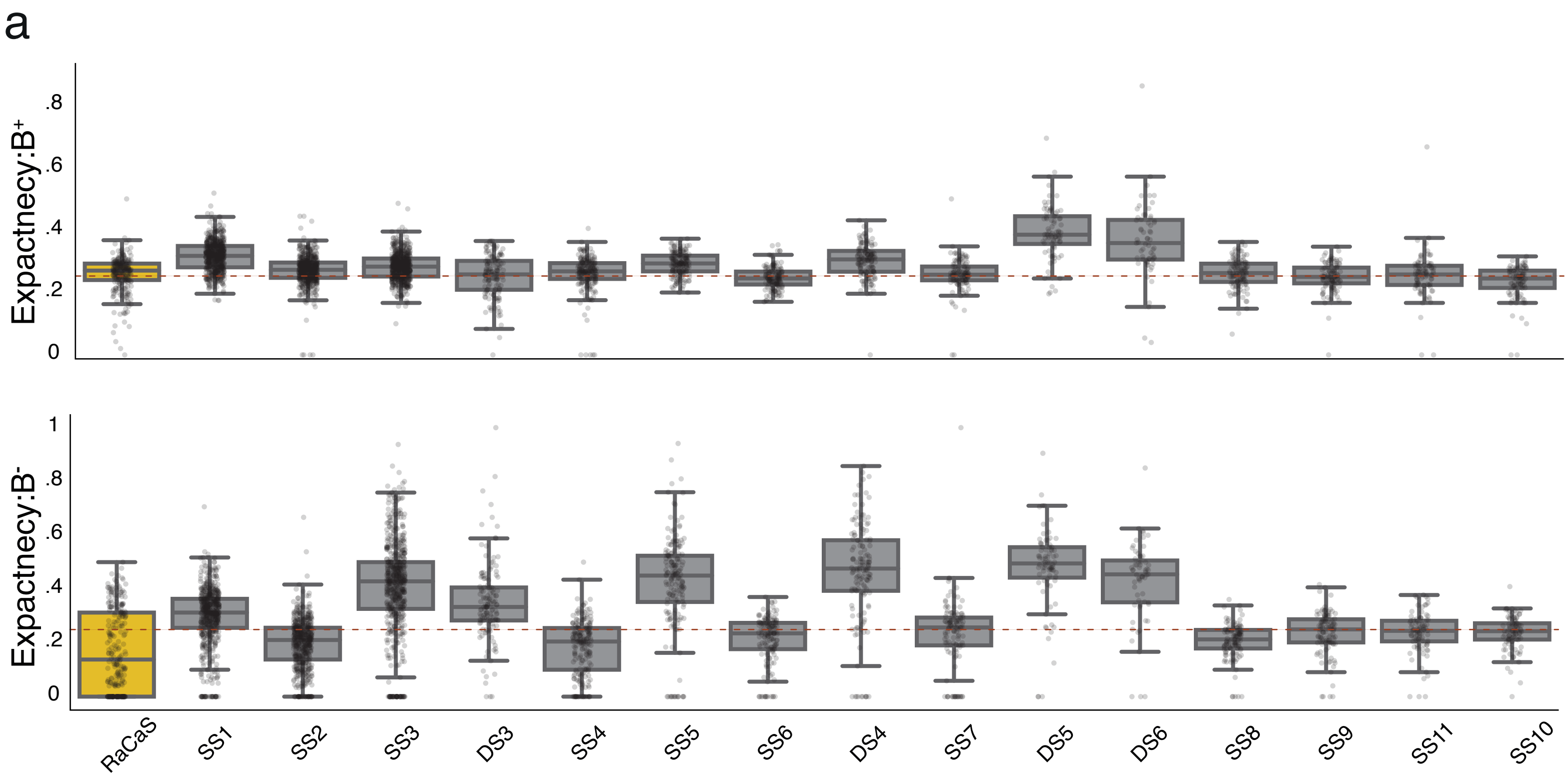

Supplementary Figure 3. *Empirically observed reward expectancy for both sides, as observed by participants assigned to RaCaS and to all other algorithms.* (A) Boxplots depicting the reward expectancy ($\frac{\#Rewards\ won(option)}{\#choices(option)}$) of all the algorithms in the CEC, ordered by magnitude of bias. RaCaS is depicted in yellow. Dots indicate individual participants. The dashed brown line indicates expectancy under random choice. Box bounds show interquartile range (IQR), line represent the median, and whiskers extend to points that lie within 1.5 IQRs.

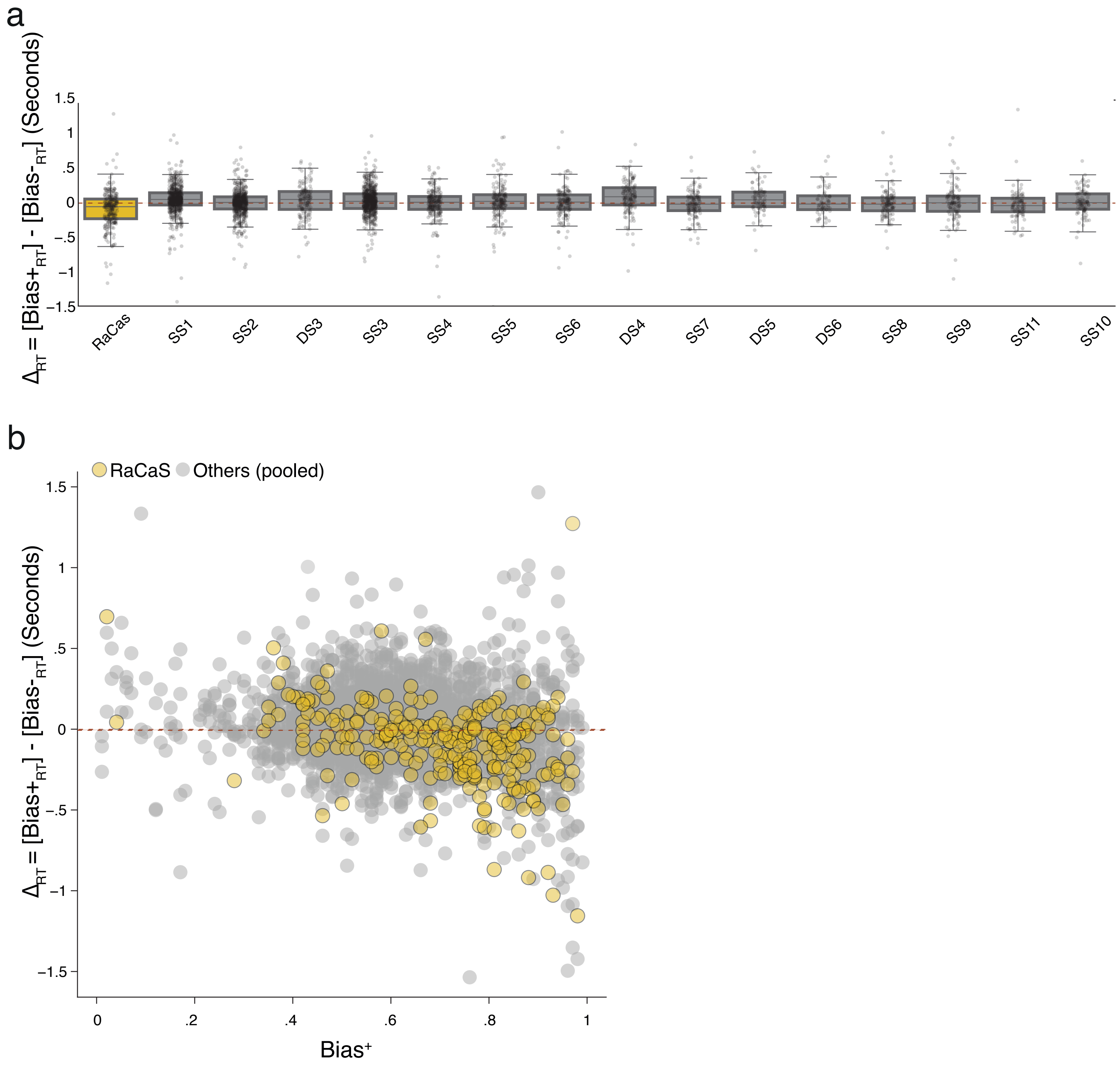

Supplementary Figure 4. *Participants in general and RaCaS participants in particular, exhibited longer response times when choosing the Bias- option; this was more pronounced for highly biased participants.* (A) Boxplots depicting the difference in Response Times (Δ_{RT}) calculated per participant, between all the trials where the Bias+ option was chosen vs. those where the Bias- option was chosen. The box plots depict all the algorithms participating in the CEC, ordered by magnitude of bias. RaCaS is depicted in yellow. Dots indicate individual participants. The dashed brown line indicates expectancy of delta under random choice. (B) Overlaid scatter plots depicting Δ_{RT} over strength of Bias+. Participants under RaCaS are depicted in yellow and those under all other competitors are depicted in grey. *RaCaS participants* show a greater difference between response times for bias + and bias -as a function of the strength of the bias. The dashed brown line indicates expectancy under random choice. Before analyzing response times, we excluded trials with RT below the minimum value technically possible (1.5 S, as stated by the CEC organizers) or more than twice the mean RT (mean=3.5). These trials constituted 3.7% of the data. Box bounds show interquartile range (IQR), line represent the median, and whiskers extend to points that lie within 1.5 IQRs.

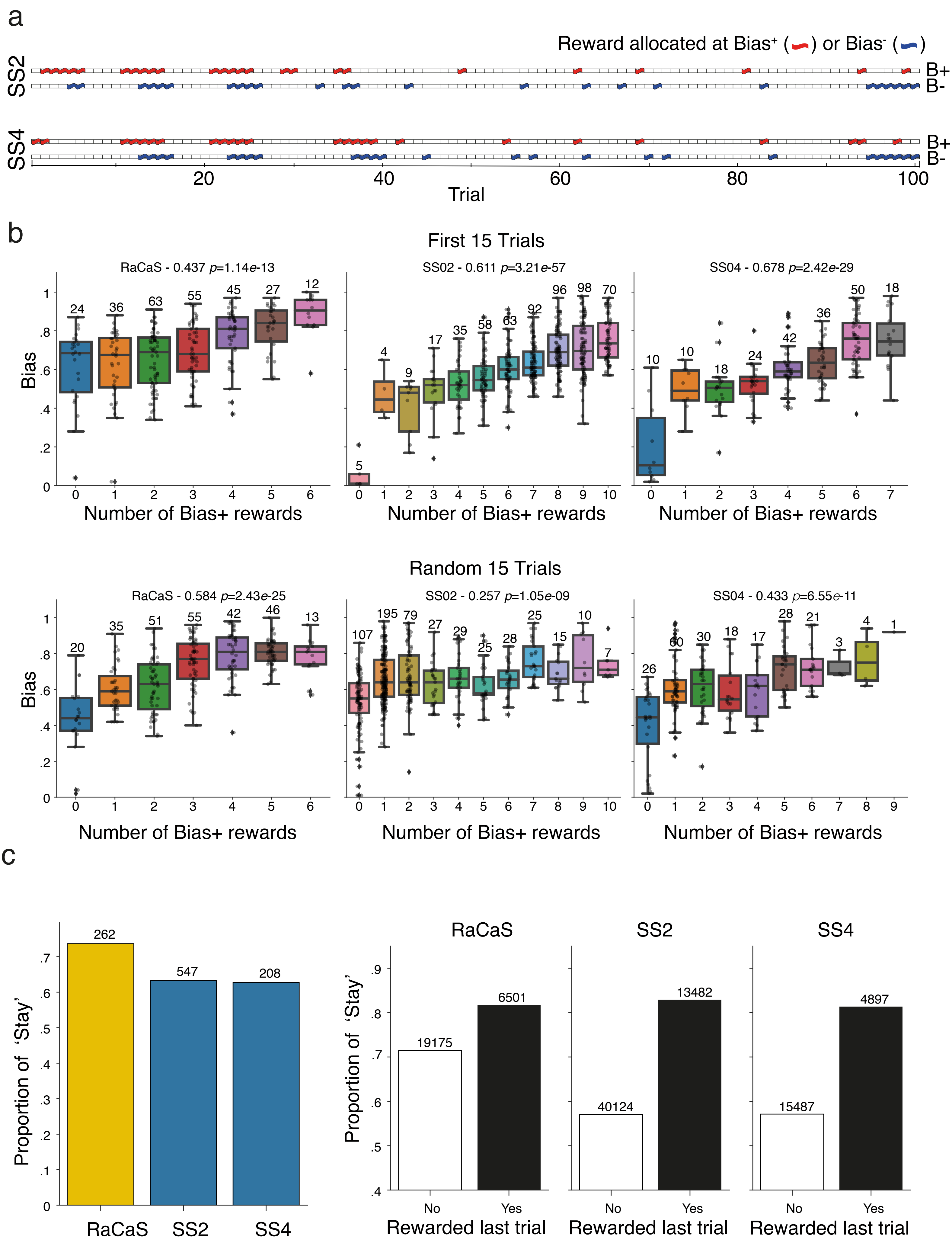
a
Reward allocated at Bias+ ( ) or Bias- ( )
SS2
SS4
B+
B-
20
40
60
80
100
Trial
b
First 15 Trials
RaCaS - 0.437 p=1.14e-13
SS02 - 0.611 p=3.21e-57
SS04 - 0.678 p=2.42e-29
Bias
Number of Bias+ rewards
Random 15 Trials
RaCaS - 0.584 p=2.43e-25
SS02 - 0.257 p=1.05e-09
SS04 - 0.433 p=6.55e-11
c
Proportion of 'Stay'
262
547
208
RaCaS
SS2
SS4
19175
6501
40124
13482
15487
4897
No
Yes
Rewarded last trial

Supplementary Figure 5. *Assessing the biasing efficacy of different elements in RaCaS and in SS2/4: "First impression matters" and "If it ain't broke, don't fix it".* (A) The deterministic schedules of reward allocation for SS2 and SS4 we submitted to the CEC. Unlike the dynamic RaCaS, rewards allocation was fixed and shared by all participants assigned to these schedules. This enables both the assessment of the efficacy of the elements employed to affect participants' choice behavior as well as use the difference of these elements' efficacy for RaCaS' participants. Such a difference will enable us to better estimate the effect of RaCaS' unique elements, most notably, its implementation of an unfolding sequence. The 3 elements designed to influence participants choice behavior in SS2/4 can be seen in the figure (see further elaboration in text): (1) 'First impression matters'. both schedules (SS2 more prominently) were designed to first reward (only) choices of $Bias^+$ (in red) (2) 'If it ain't broke don't fix it'. both schedules allocated rewards to $Bias^+$ in continuous sequences. Under the same principle. rewards obligatorily allocated to $Bias^-$ (in blue) were 'hidden' by lagging them slightly after the initiation of a reward sequence in $Bias^+$ (3) 'Learned helplessness'. Further rewards allocated to Bias+ were assigned to the final trials of the experiment under the assumption that given 1 & 2, when reaching these trials, participants will have acquired a sufficient number of failures to obtain rewards in $Bias^-$ to stop them exploring that option altogether and thus missing out on the late trove of rewards. (B) Empirical evidence for the "First impression matters" element in SS2/4 and in RaCaS. As can be seen, the correlation between the sum of rewards obtained in the first 15 trials and total bias is substantial under all 3 schedules, although significantly lower in RaCaS compared to the 2 static schedules. The lower row presents a benchmark for testing the unique effect of rewards obtained in the first 15 trials by showing the correlation between total bias and a random sample of trials between trial 16 and 100. As can be seen, the correlations in both SS2/4 are significantly lowered, but increases for RaCaS, the correlation. These results show that rewards affect choice behavior differently in the static schedules and in RaCaS. All $p<.001$ (C) Empirical evidence for "If it ain't broke, don't fix it" element in SS2/4 and in RaCaS. The left figure depicts the general proportion of 'Stay' decisions in all 3 schedules (calculated over all participants, where each participant contributes 99 observations to the pool), with RaCaS leading to a larger proportion of trials in which participant stayed with the previously chosen option; Error bars indicate +/- standard errors. The right figure shows the 'Stay' proportion, now conditioned on whether the former choice was rewarded or not. While being rewarded on the previous trial leads to an increase in the proportion of staying with the (rewarded) option, this effect is visibly smaller for RaCaS, further suggesting that immediate rewards have a muted effect under RaCaS. Here, means are calculated across observations and no error bars are presented. Box bounds show interquartile range (IQR), line represent the median, and whiskers extend to points that lie within 1.5 IQRs.

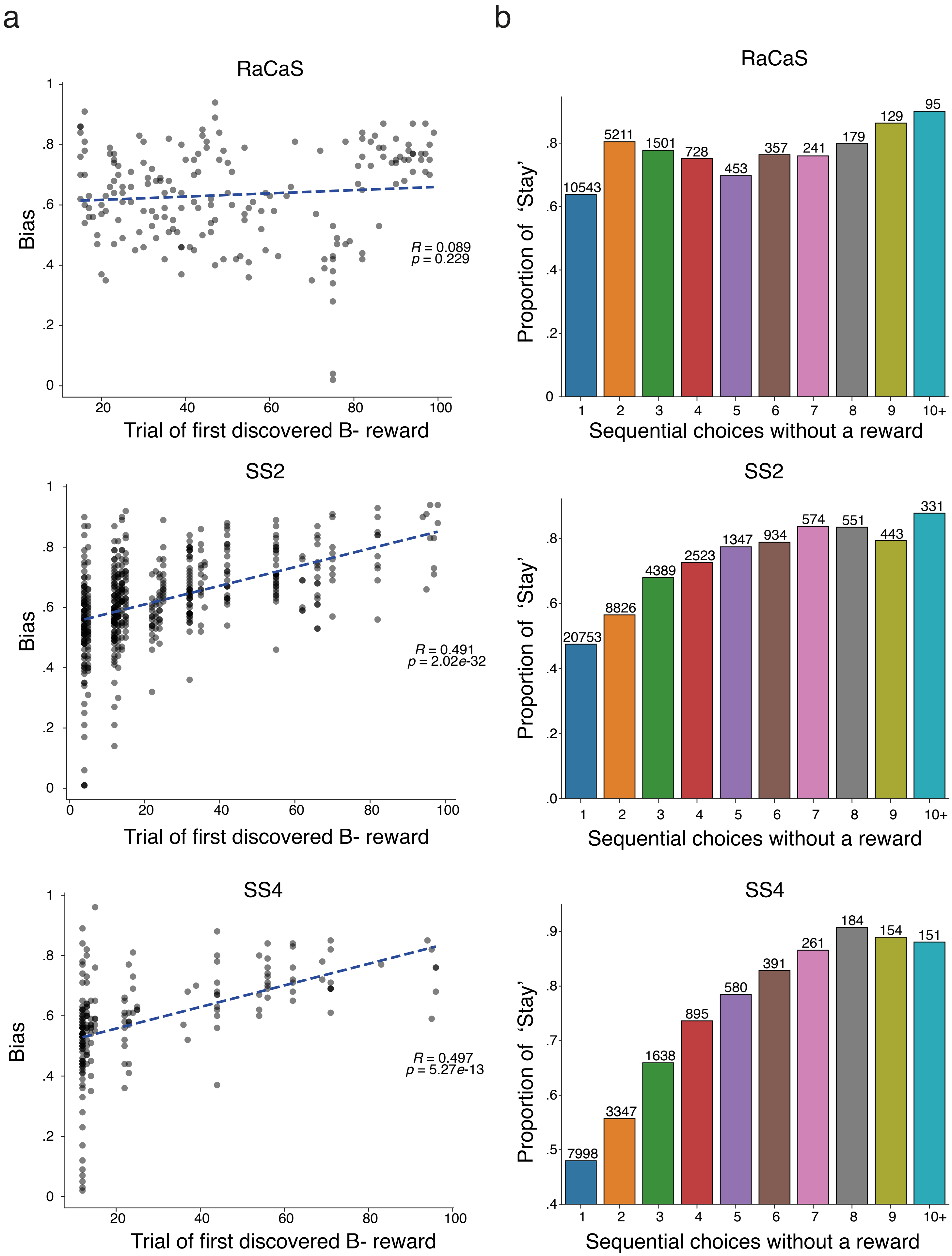
a
RaCaS
Bias
Trial of first discovered B- reward
R = 0.089
p = 0.229
SS2
Bias
Trial of first discovered B- reward
R = 0.491
p = 2.02e-32
SS4
Bias
Trial of first discovered B- reward
R = 0.497
p = 5.27e-13
b
RaCaS
Proportion of 'Stay'
Sequential choices without a reward
10543
5211
1501
728
453
357
241
179
129
95
SS2
Proportion of 'Stay'
Sequential choices without a reward
20753
8826
4389
2523
1347
934
574
551
443
331
SS4
Proportion of 'Stay'
Sequential choices without a reward
7998
3347
1638
895
580
391
261
184
154
151

Supplementary Figure 6*. Assessing the biasing efficacy of different elements in RaCaS and in SS2/4: "Learned helplessness" and "The gamblers fallacy".* (A) Empirical evidence of the "Learned helplessness" element. Spearman correlations and regression lines relating total bias and time (trial) of first rewarded $Bias^-$ trial. As can be seen this correlation is sizeable for SS2 and SS4 suggesting that hiding the rewards in $Bias^-$ effectively led to a stronger bias towards $Bias^+$. The same relationship is practically non-existent in RaCaS, yet again showing that the mere allocation of rewards was far less important as compared to the $r(511) = 491$, $p < .001$; SS4 – $r(184) = .497$, $p<.001$(B)

The relationship between the number of sequential unrewarded selection of the same option and the proportion of switching. Surprisingly, in both SS2 and SS4, the longer an unrewarded sequence of selection of an option was, the lower was the probability of switching away from it. A potential case of Gambler's fallacy driven choice-behavior. Notably, in RaCaS, the above pattern only appears after crossing the maximal number of unrewarded trials afforded by the regular sequence.

Supplementary References

1. Dan, O. & Loewenstein, Y. From choice architecture to choice engineering. *Nature Communications* vol. 10 (2019).
2. Dan, O., Plonsky, O. & Loewnestein, Y. Towards Choice Engineering. *bioRxiv* 2023.11.04.565653 (2023) doi:10.1101/2023.11.04.565653.
3. Glimcher, P. W. Understanding dopamine and reinforcement learning: The dopamine reward prediction error hypothesis. *Proc. Natl. Acad. Sci. U. S. A.* **108**, (2011).
4. Edwards, W. Probability learning in 1000 trials. *J. Exp. Psychol.* **62**, (1961).
