# Supplementary material for: Using an algorithmic approach to shape human decision-making through attraction to patterns
Source: Nat Commun. 2025 May 2;16:4110. doi: 10.1038/s41467-025-59131-4 (PMC12048589; doi:10.1038/s41467-025-59131-4)
Supplement: Supplementary file 2 — Description of Additional Supplementary Files [file 41467_2025_59131_MOESM2_ESM.pdf]

Description of Additional Supplementary Files

File Name: Supplementary Data 1

Description: Full results and statistics. All statistical tests are one-sample t-tests, conducted for each model against the result expected under H_0 (represented by a brown dashed line in all figures). DS01 and DS02 are the models that constitute RaCaS. Other than these two exceptions, schedules are sorted by the bias they induced. DS - Dynamic Schedule. SS - Static Schedule. P-values limits are presented up to $P < 10^{-8}$.
